# A case report of Gollop-Wolfgang complex in 12 years old boy^☆^

**DOI:** 10.1016/j.ijscr.2023.108223

**Published:** 2023-04-15

**Authors:** Ahmad Elewee, Wafik Mayo, Bashar Mirali, Mohammad Ezzat Alaktaa, Osama Hmaidy

**Affiliations:** a Department of Orthopedic Surgery, Damascus Hospital, Damascus, Syria; b Faculty of Medicine, Aleppo University, Aleppo, Syria; c President of Department of Orthopedic Surgery, Damascus Hospital, Damascus, Syria; d Department of Urologic Surgery, Faculty of Medicine, Damascus University, Damascus, Syria

**Keywords:** Case report, Gollop-Wolfgang complex, Distal bifid femur, Tibial agenesis

## Abstract

**Introduction:**

The Gollop-Wolfgang Complex (GWC) was initially described by Gollop et al. and is a rare congenital limb anomaly disorder characterized by the association of distal bifid femur and tibial agenesis.

**Case presentation:**

This study presents a case of a 12-year-old boy with Gollop-Wolfgang Complex (GWC), a rare congenital limb anomaly disorder characterized by the association of distal bifid femur and tibial agenesis. The patient did not have any VACTERL abnormalities and had a normal level of intelligence. Examination revealed coxa valga in both hips and upper limbs on both sides, a shortened left leg with a palpable bony protuberance and absence of the patella, and a shortened right leg with a palpable fibula lateral to the knee and absent tibia with severe knee varus deformity on both sides. Both feet revealed equinovarus deformity with ectrodactyly. The patient underwent through-knee amputation and was fitted with two prostheses to provide enhanced functional support.

**Clinical discussion:**

The etiology of GWC is still unknown, but errors in the complex genetic control of limb development are believed to be related.

**Conclusion:**

Treatment choice depends on the deformity type, with through-knee amputation recommended for cases with observed flexion contracture, bifid femur, and tibial hemimelia, followed by modern prosthesis fitting for optimal outcomes. This case illustrates the efficacy of this surgical management and highlights the need for ongoing follow-up care.

## Highlights

• Gollop-Wolfgang Complex is a rare congenital limb anomaly disorder characterized by the association of distal bifid femur and tibial agenesis.
• GWC has been associated with various congenital abnormalities in the other organs included within the VACTERL sequence.
• An autosomal dominant inheritance pattern within complete penetrance is the most common mode of GWC.
• Tibial aplasia was divided into type Ia and Ib regarding the radiographic findings.
• Through-knee amputation is recommended for trans-osseous amputation in pediatrics.
• We report a 12-year-old boy with the GW complex with a follow-up of 4 months following surgical management.

## 1 Introduction

Gollop-Wolfgang Complex (GWC) is a rare congenital limb anomaly disorder characterized by the association of distal bifid femur and tibial agenesis. The condition was initially described by Gollop et al. in 1980 in patients with unilateral femoral bifurcation, bilateral absence of tibia, monodactyly of the feet, and ectrodactyly of one hand [1]. In 1984, Wolfgang reported a case of right femoral bifurcation, tibia hemimelia, and contralateral diastasis of the tibia [2]. Moreover, femoral bifurcation and hand ectrodactyly were excluded from the Gollop-Wolfgang Complex in 1986 by Lurie and Ilyina [3].

GWC has been associated with various congenital abnormalities in the other organs included within the abbreviation (VACTERL) (vertebral defects, anal atresia, cardiac defects, tracheoesophageal fistula, renal anomalies, and limb abnormalities) sequence [4]. An autosomal dominant inheritance pattern within complete penetrance is the most common mode, with other observed patterns including X-linked and autosomal recessive. Neomutations could also exist through the reported cases [4].

The skeletal structure of the tibia determines surgical management. Tibial aplasia was divided into type Ia and Ib regarding the radiographic findings by Jones et al., which demonstrate complete tibial absence with or without a hypoplastic lower femoral epiphysis, respectively. The extensor mechanism in the affected leg guides the surgical management with preferred knee disarticulation for the treatment of Jones type I. To avoid overgrowth in pediatrics, through-knee amputation (disarticulation) is recommended for trans-osseous amputation. However, limb salvage procedures are recommended for those with functioning extensor mechanisms without significant fixed knee flexion contracture [5].

The etiology of GWC is still unclear, but it is most likely postulated to be an error in the complex genetic control of limb development [1]. Regarding the United States Office of Rare Diseases [ORD] of the National Institute of Health [NIH], GWC is classified as a “rare disease” with fewer than 200 reported cases to date. In addition, it has an incidence of 1 in 1000,000 live births [6].

In this case, we report a 12-year-old boy with the GW complex with a follow-up of 4 months following surgical management. It illustrates the efficacy of the through-knee amputation and appropriate two prostheses to provide enhanced functional support to the patient. Informed consent was obtained from the parents regarding the publication of this manuscript. We have checked our case by SCARE guidelines [7].

## 2 Presentation of case

A 12-year-old male Syrian child with normal karyotype (46 XY) was born at 39 weeks of gestation after a normal pregnancy and delivery to a 28-year-old primigravida/G8P6 mother.

No history of maternal drug ingestion, alcohol, tobacco, illicit drug, diabetes mellitus, or infection was reported. No parental consanguinity was present and family history was negative for any birth defects. Family history was negative for any congenital abnormalities (VACTERL association) or limb deficiencies.

The child was breastfed with good cry and appetite, without any change in skin color, bowel bladder problems, or cleft lip/palate. Ultrasonography of pelvis and abdomen revealed no renal or visceral abnormalities. Echocardiography at the time of admittance revealed no congenital heart defects. The child had a normal level of intelligence. Prenatal diagnosis was not available due to lack of information and resources with the family. We have explained that in the manuscript as well.

**Fig. 1:**
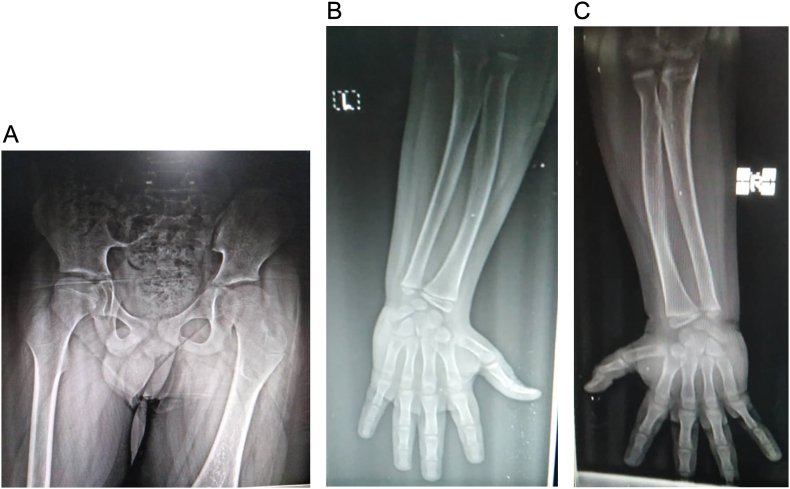
Normal hip (A) and normal left and right upper limbs (B and C).

**Fig. 2:**
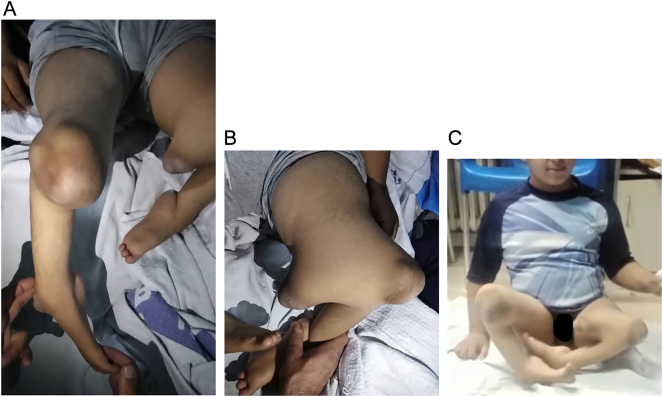
Left bifid femur (A) with the absence of left patella, severe knee varus deformity, and ipsilateral complete tibial hemimelia (A, B, C and Video 1). Left bifid femur (A) with the absence of left patella, severe knee varus deformity, and ipsilateral complete tibial hemimelia (A, B, C and Video 1).

On physical examination, there has been coxa valga in both hips, while the upper limbs were normal (Fig. 1-A and B). A flexion contracture of both knees was observed with no mobility of the quadriceps mechanism. The left leg was shortened with a palpable bony protuberance in the medial thigh proximal to the knee joint, with the absence of the Patella. The lateral branch articulated the fibula suggesting an immobile knee joint (Fig. 2-A and C). Examination of the right leg revealed a shortened leg with a palpable fibula lateral to the knee. The tibia was absent with severe knee varus deformity on both sides (Fig. 2-B and C). Both feet also revealed equinovarus deformity with a small width. An ectrodactyly was observed in both feet of hallux on the right foot, and hallux and 2nd toe on the left foot (Fig. 2-A, B, and C).

**Fig. 3:**
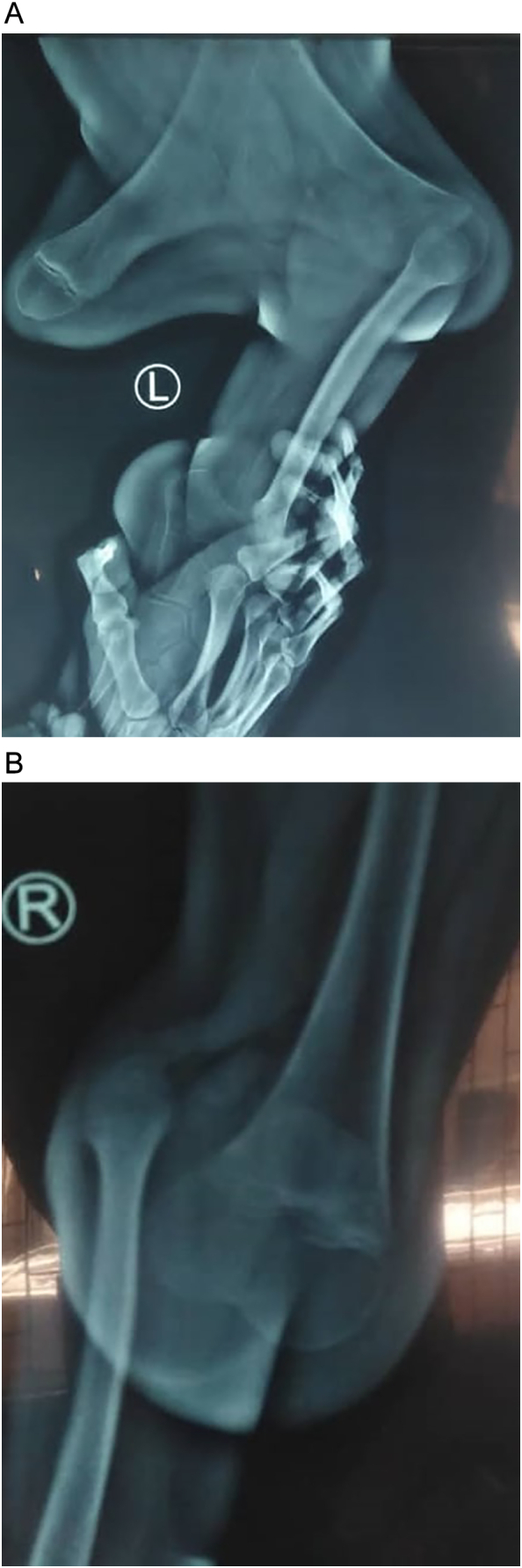
X-ray image of the left bifid femur and complete tibial hemimelia (A) and complete tibial hemimelia of the right limb (B).

**Fig. 4:**
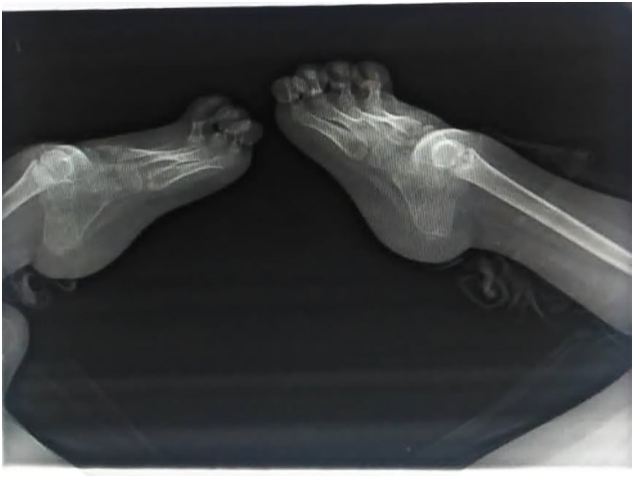
Absence of hallux and 2nd toe on the left foot and of the hallux on the right foot.

Roentgenograms demonstrated the physical lower extremity findings, which were consistent with GWC including left bifid distal femur with the absence of left patella and complete tibial hemimelia (Fig. 3-A and B). Radiographic images showed absence of the metatarsal and phalanges bones of the hallux and 2nd toe in the left foot and hallux of the right foot (Fig. 4).

**Fig. 5:**
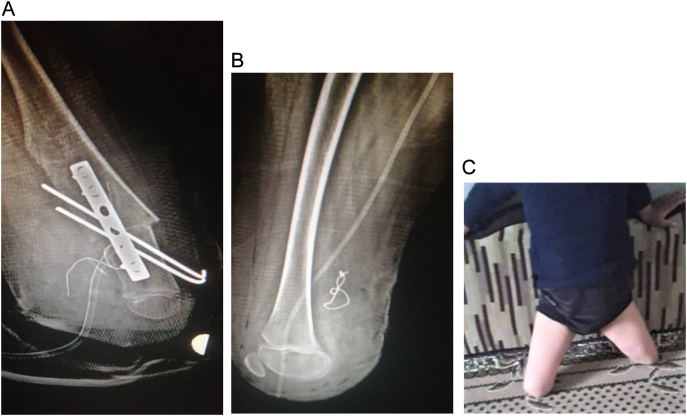
Left and right limbs after the surgical reconstruction (A, B, and C).

**Fig. 6:**
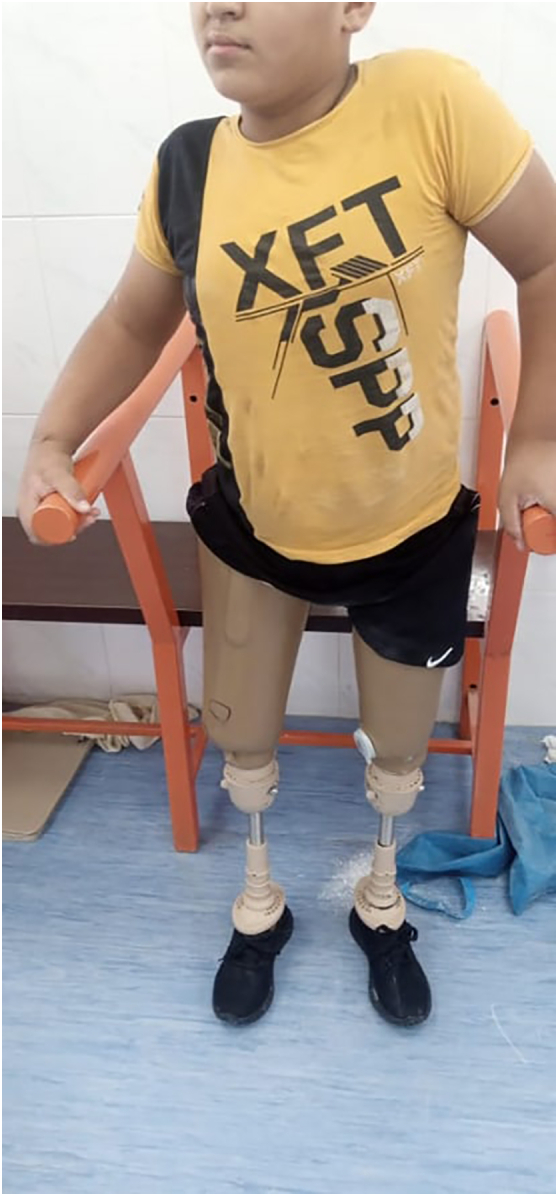
Patient walking independently 2 years following surgical reconstruction wearing right and left lower extremity prosthesis.

Lower extremity reconstruction was discussed with the patient's parents to promote amputation after prosthetic fitting. Both lower legs were surgically ablated (through-knee amputation) with the removal of the non-functional medial protrusion of the left femur in the poorest alignment. Throughout the surgery, the mechanical and anatomical axis of the 50 femoral valgus were corrected with the protection of the femoral artery (Fig. 5-A, B, and C). Finally, a rigid well-padded dressing was placed to secure the hip (Fig. 6).

The child was scheduled for 3 months in a physical rehabilitation program. Once his residual limb was stabilized and the wound was well healed, two above-knee prostheses were fitted. Half a year later the child was able to walk independently with two above-knee prostheses. At his one-year follow-up visit, he was 14-year-old, and the radiographic findings showed a healed osteotomy with preserved distal and proximal femoral physis (Video 2).

## 3 Discussion

Tibial hemimelia almost always occurs in combination with bifid femur which are extremely rare anomalies with an incidence of lower than 1 in 1,000,000 live births [6]. This pattern of defects was explained in detail by Wolfgang in 1984 [8]. Tibial agenesis (tibial hemimelia) is a congenital deformity involving anomalies of both ankles, knee joints, and the adjacent musculotendinous units [9]. Bifurcation of the femur (bifid femur) is described as a more or less severe bowing of the upper leg which could be associated with equinovarus deformity of the foot or knee and patella absence [10]. This association usually manifests with other congenital anomalies of the limbs as in the ectrodactyly of the hand or feet, or other parts of the body as in VACTREL syndrome [4]. The term Gollop-Wolfgang complex is used to describe the association of bifid femur, tibial hemimelia, and feet ectrodactyly with or without hand ectrodactyly as in our case.

This case study presents a 14-year-old patient with a diagnosis of Gordon-Weber-Cockayne Syndrome (GWC), characterized by absent patella, hand ectrodactyly, and normal hip and upper limb manifestations. Additionally, the patient exhibited severe equinovarus in both the right knee and foot, as well as flexion contracture in both knees. No information was available regarding the parents' decision to terminate the pregnancy or not, as the routine prenatal counseling and care were provided by another hospital.

Two distinct developmental fields are controlling the growth of the lower limb; the tibia and fibula, according to Lewin and Opitz hypothesis. The tibial developmental field defects control the development of the distal femur, tibia, and hallux. Thus any defect in it results in a bifurcation of the distal femur, tibial hemimelia, and preaxial ectrodactyly or polydactyly, occasionally in association with hand ectrodactyly. The fibular developmental field defects include the pubic bones, the proximal femur, the patella, the fibula, and the lateral rays of the foot. A defect in this field leads to proximal focal femoral deficiency, fibular hypoplasia, ectrodactyly, and any deficiency of the lateral knee ligament [11]. Moreover, there is a noticeable association between both development fields [4]. Our patients showed a defect in the right lower limb tibial field with an absent tibia, ectrodactyly of the hallux. Nevertheless, both the tibial and femoral fields were defective in the left lower limb, resulting in the absent patella, medial bifid femur, absent tibia, and ectrodactyly of both hallux and 2nd toe.

Forzano et al. have stated that tibia agenesis-ectrodactyly syndrome was the only differential diagnosis regarding the Gollop-Wolfgang complex [12]. Moreover, several additional internal malformations in association with bifid femur and tibia agenesis have been discussed in the medical literature. Cardiac defects have been noticed in infants with GWC [13]. Raas-Rothschild et al. [13] and van de Kamp et al., [14] have discussed its association with VACTREL syndrome [13], [14]. Evans and Chudley reviewed eight cases of patients with GWC and caudal midline defects as well as their case [15]. All of the mentioned cases lacked any involvement of the upper limb, which indicates the localizing of the defect to the caudal region.

Several possible causes regarding the etiology were discussed in the medical literature. Many of them have suggested a genetic defect [15], [16], while others discussed the effect of carbamazepine [17] and valproic acid [18] as risk factors for the GWC. Elucidation of the main etiology requires a broad analysis that includes all cases published in the medical literature, with a comparison of the family and genetic history of each.

Treatment of the tibial hemimelia was classified in three systems; Jones, Kalamchi and Dawe, and the newer web classification [4]. The most commonly used was the Jones classification which has divided it into four types; type 1: the tibia is absent at birth on radiographs. With two different subtypes; 1a the tibia is completely absent and the ossific nucleus of the distal femoral epiphysis is hypoplastic and 1b: the proximal part of the tibia is present, but unossified at birth, hence appears absent on plain radiograph. In this type, there is normal ossification of the distal femoral epiphysis. Type 2: the proximal part of the tibia is ossified and visible on radiographs at birth, but the distal tibia is absent, type 3: the distal part of the tibia is ossified and visible, but the proximal portion of the tibia is absent. This is the least common type of tibia hemimelia, and type 4: the tibia is short, and there is distal tibiofibular diastasis [4], [19]. Treatment options depend on the type of defect. In type 1; as in our case, it is preferred to resection the arm of the bifid femur, osteotomy, and re-alignment of the rest with knee disarticulation, in addition, a prosthetic fitting to improve function [5].

## 4 Conclusion

There are several recommendations throughout the discussion of our case management and the medical literature. Regarding the etiology of GWC, it is crucial to look for it in mothers with similar family history or carbamazepine and valproic acid intake during pregnancy, as the medical termination of pregnancy could be discussed with the family. When it is detected, genetic counseling is advised, and the patient should be thoroughly clinically and radiologically checked for concomitant abnormalities. Treatment choice depends on the deformity type, with recommended through-knee amputation for cases with observed flexion contracture, bifid femur, and tibial hemimelia followed by a modern prosthesis fitting to achieve the best course of action.

The following are the supplementary data related to this article.

Video 1 The patient after the surgical intervention and the installation of the prosthesis. Video 1

Video 2 Patient walking independently 2 years following surgical reconstruction wearing right and left lower extremity prosthesis. Video 2

## Consent

A Written informed consent was obtained from the patient's parents for publication of this case report and accompanying images. A copy of the written consent is available for review by the Editor-in-Chief of this journal on request.

## Ethical approval

We have an ethical approval from the ARB in University of Damascus.

## Funding

N/A.

## Author contribution

Wafik Mayo: writing

Osama Hmaidy: Data collecting

Bashar Mirali and Mohammad Ezzat Alakataa: supervisors of the surgical procedure

Ahmad Elewee; the main surgeon who diagnosed the case and conducted the surgical procedure

## Guarantor

Wafik Mayo.

Osama Hmaidy.

Ahmad Elewee.

Take full responsibility.

## Research registration number

Not applicable.

## Declaration of competing interest

There was no need for funding.

## Data Availability

The data that support the findings of this study are available from the corresponding author upon reasonable request.
